# Does design change in total knee arthroplasty implants affect patient-reported outcomes?

**DOI:** 10.1186/s12893-023-01948-1

**Published:** 2023-03-07

**Authors:** Nader Toossi, Brandon Bucklen, Lindsey K. Meding, John B. Meding

**Affiliations:** 1 Musculoskeletal Education and Research Center (MERC) Audubon, 2560 General Armistead Ave, Audubon, PA 19403 USA; 2 The Center for Hip and Knee Surgery, St. Francis Hospital Mooresville Mooresville, 46158 Mooresville, IN USA

**Keywords:** Total knee arthroplasty, Patient-reported outcome, KOOS-JR, Knee society score

## Abstract

**Purpose:**

The purpose of this study is to compare the early results of patient-reported outcomes between two generations of a total knee system.

**Methods:**

Between June 2018 and April 2020, 121 first-generation, cemented TKAs (89 patients) and 123 s-generation, cemented TKAs (98 patients) were performed by a single surgeon. Demographic and surgical data were collected from all patients. Starting at the 6-month follow-up, patient-reported outcome measures Knee Injury and Osteoarthritis Outcome Score, Joint Reconstruction (KOOS-JR) and Knee Society (KS) clinical and radiographic scores were prospectively recorded. This study represents a retrospective review of these prospectively collected data.

**Results:**

There were no statistically significant differences between the two groups in terms of demographic variables such as age, body mass index, gender and race. KOOS-JR and Knee Society (KS) scores improved significantly (p < 0.001) from their preoperative values in both device generations. There were no differences, pre-operatively, between the two groups in terms of KOOS-JR, KS functional, KS objective, patient satisfaction, and expectation scores; however, there were statistically significant (p < 0.001) lower values of KOOS-JR and KS functional scores for first versus second generation at 6 months (81 vs. 89 and 69 vs. 74, respectively).

**Conclusion:**

While significant improvement in KS objective, subjective, and patient satisfaction scores were noted with both knee systems, KOOS-JR and KS function scores were significantly higher at the early (6-month) follow-up in the second-generation group. Patients responded acutely to the design change as evidenced by significantly improved patient-reported outcome scores for the second generation.

## Introduction

Total knee arthroplasty (TKA) has been one of the most successful procedures in orthopedics [1]. However, it is not without complications [2]. Some of these complications are related to the implant itself, while others such as periprosthetic joint infection and stiffness are inherent to the procedure and host factors [3, 4]. Manufacturers have designed different implants over the last couple of decades to address some of the main complications.

One of the original implants that had gained popularity among surgeons was the Insall-Burstein (IB) knee system, which was developed in 1978 [5]. The first two generations of IB implants were known for the increased rate of patellofemoral problems [6–8]. The PROVEN (1G: 1st Generation) total knee system from StelKast (Globus Medical, Audubon, PA, USA), could be viewed as the third generation of IB knees addressing some of the patellofemoral issues encountered with previous IB knee systems [9]. The next generation total knee system, GenFlex_2_™ (2G: 2nd Generation) was introduced to the market recently.

Although patient-reported outcomes (PRO)s following primary TKA were the subject of many published studies, little has been reported regarding the effect of design changes on these outcomes. The purpose of this study was to compare patient-reported outcomes of 1G TKA with those of 2G TKA focusing on the technical design differences between the two platforms.

## Methods and materials

This was a retrospective cohort study on prospectively collected data of a sample of consecutive patients undergoing total knee arthroplasty due to end-stage osteoarthritis unresponsive to conservative treatments at a single facility by a fellowship-trained joint reconstructive surgeon. The study period was between June 2018 and March 2021. Institutional Review Board (IRB) exemption was obtained prior to study initiation. Waiver of informed consent was issued by the same IRB. There were 89 patients (121 knees) treated with 1G and 98 patients (123 knees) treated with 2G who consented to be enrolled for the study. The surgeon switched from 1G to 2G prostheses once the new implants were available to order. No changes in patient selection strategies were made once the new generations were implanted. Patients were excluded from the study if they had a history of metabolic bone disease (such as Paget’s disease of bone, severe osteoporosis), systemic conditions affecting bone density (e.g. renal osteodystrophy; inflammatory arthritis), bony defects requiring grafting, a poorly functioning contralateral TKA or revision regardless of function.

All TKAs were performed via a medial parapatellar approach using an intramedullary femoral alignment guide set at five-degrees and an extramedullary tibial alignment guide set at neutral in the coronal plane with a neutral posterior slope in the sagittal plane. All TKAs were cemented (Palacos^®^, Heraeus Medical, Hanau, Germany). The postoperative protocol was the same in all cases including deep vein thrombosis prophylaxis, prophylactic antibiotics, and follow-up schedule (8 weeks, 6 months, 1 year, and every 1 to 2 years thereafter). Physical therapy was initiated on the day of operation. Each exam was performed by the attending physician.

As per the study protocol, the surgeon switched from 1G to 2G a year into the study period. Data for demographic parameters including age, gender, race, and body mass index (BMI) were collected preoperatively. Scores from patient-reported outcome measures such as the Knee Injury and Osteoarthritis Outcome Survey-Joint Replacement (KOOS-JR) [10] and Knee Society clinical and radiographic scoring system (KSS) were collected at each office visit [11]. Scores from different components of KSS were reported separately. These components were objective knee score, functional score, patient satisfaction and expectation score. Intra- and post-operative complications, as well as any revisions, reoperations, and returns to operating room, were diligently recorded. All data were collected prospectively in an institutional database. This study represents a retrospective review of these prospectively collected data.

### Implants

Timothy Wright, PhD, from Hospital for Special Surgery, New York, NY, assisted in the design of 1G posterior stabilized implant, which is technically an evolutionary modification of IB-II. Unlike IB-II, the 1G system has cruciate retaining (CR) in addition to posterior stabilized (PS) implants. The CR implant was designed and developed by StelKast.

The feedback from 1G users and the results from explanted components after revision retrieval paved the way for a design change evaluated by a validated computational finite element model analysis. Also, 1G PS implant had a symmetric patella flange whereas the CR implant had an anatomic patella flange, and the options of femoral sizes were limited to a few in 1G knee system.

The new design has created more tibio- and patello-femoral contact areas for the PS and less for the CR implants. These changes have resulted in a more consistent range of contact areas in the 2G system between CR and PS implants, leading to a more similar wear pattern between the two.

**Fig. 1:**
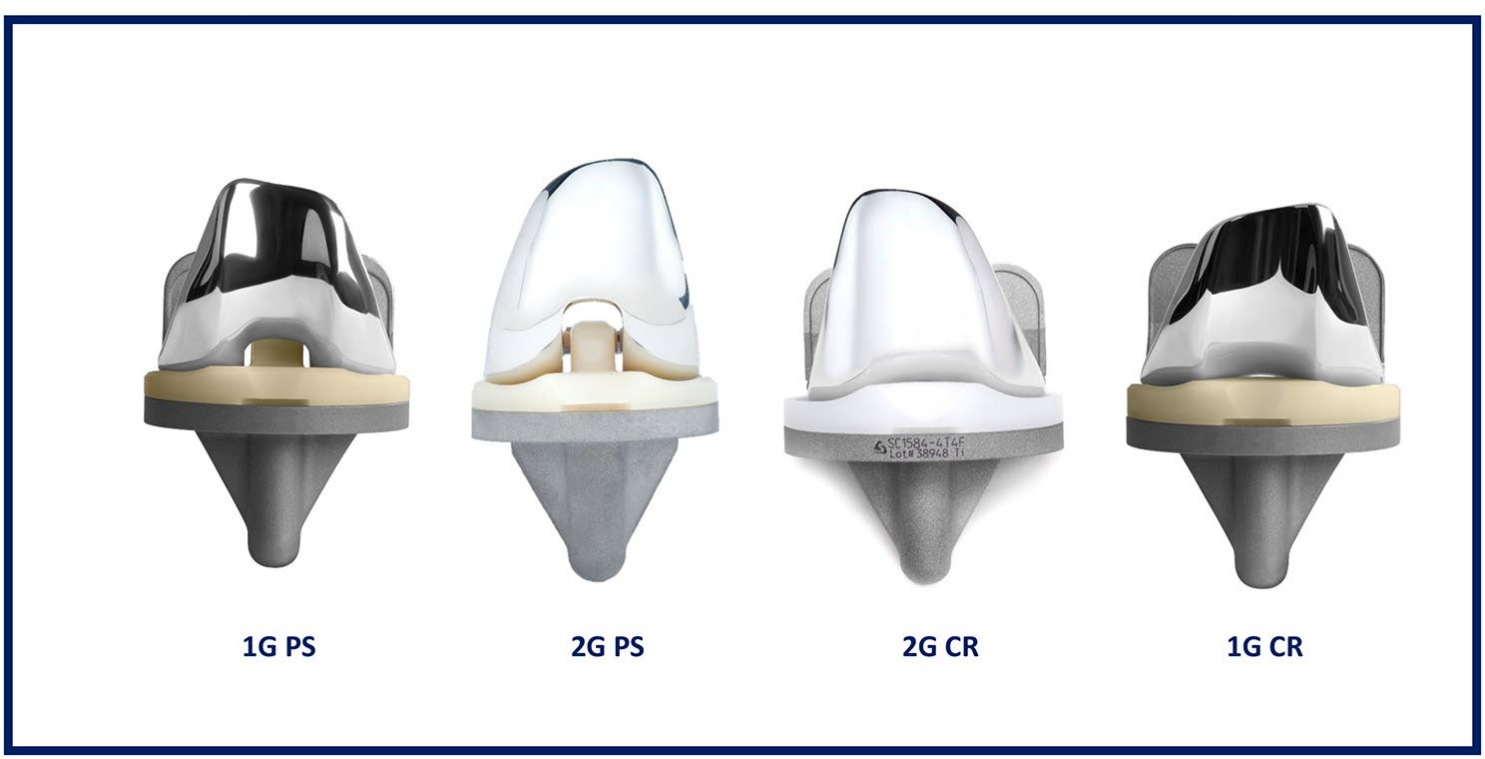
1G and 2G tibial and femoral components (PS and CR) and the corresponding inserts

**Fig. 2:**
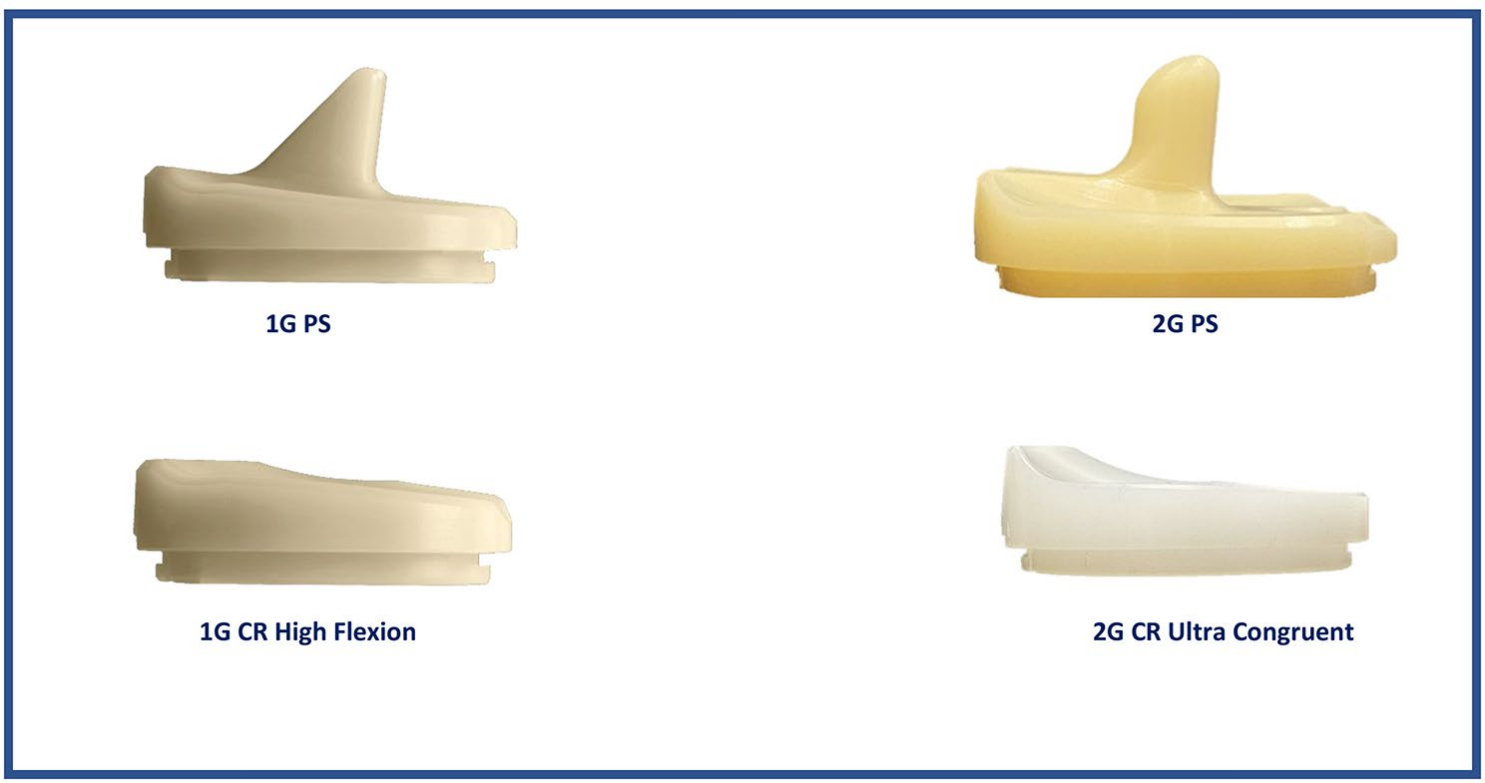
1G and 2G tibial inserts used in this study

**Fig. 3:**
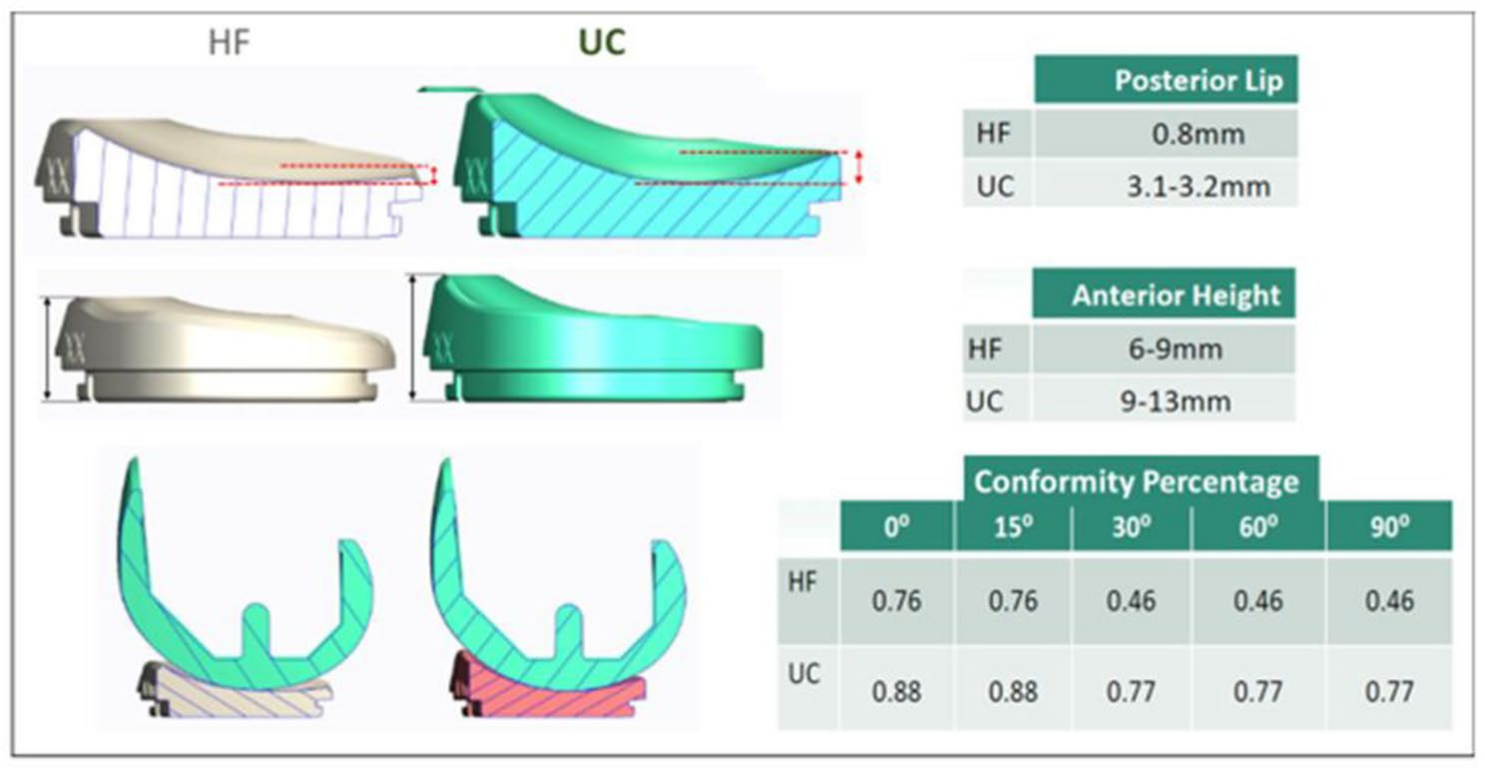
Comparison between UC and HF inserts. Conformity percentage has been measured at various degrees of knee flexion. The highest conformance is between full extension and 15° of knee joint flexion HF = High Flexion; UC = Ultra Congruent

The following changes have been made to the 1G system femoral component: debulking, implementing an asymmetric patellar flange and trochlear groove, reducing the medial-lateral profile of the anterior flange, reducing the posterior condyle length, and modifying to a trapezoidal anterior-posterior profile. For the tibial inserts, the changes included adding patellar tendon relief, implementing a round rather than a pointed post for the PS insert, and reducing the posterior lip (Figs. 1 and 2). The posterior slope of the CR insert was increased to 6°. Two different inserts, high flexion (HF) and ultra-congruent (UC), are available for the new CR implants. The UC insert is designed to offer greater conformance to the femur in the sagittal plane, an increased posterior lip height, resistance to subluxation, and an elevated anterior wall height relative to HF insert (Fig. 3). All inserts used with CR 2G in this study were UC, and were HF for 1G. The design changes were intended to improve the function of 2G over that of 1G.

All statistical analyses were performed by SPSS statistical software (version 20, IBM, Armonk, NY, USA). Independent two-sample t-tests were used to compare the means between two groups with continuous data. A Chi-squared test was used to compare two frequencies between the two groups. A p-value ≤ 0.05 was considered to be significant. For the cases lost to follow up, the latest available follow-up data were used to determine the outcome at that point. Based on a power analysis to determine the sample size, it was found that If the true difference between the means of the two groups was 2.1(the minimum difference found between average PRO scores), we would need to study 65 subjects in each group to be able to reject the null hypothesis that there was no difference between the groups with a probability (power) of 0.8. The Type I error probability associated with testing the null hypothesis was 0.05.

## Results

There were no significant statistical differences between the two cohorts in terms of age, gender, BMI, race or health status as assessed by American Society of Anesthesiologists (ASA) physical status classification system (Table 1). Out of 121 1G knees, 104 were assessed at 6 months and 89 at the 1-year follow-up visit. There were 97 2G knees evaluated at 6 months and 76 at the 1-year follow-up visit. All the knees had the patella resurfaced. One hundred and one knees in the 1G cohort (83%) and 109 (88%) in the 2G had CR implants (p = 0.24).

**Table 1:** Demographic Data

	1G	2G	p-value
Average Age (years) (range)	68.45 (41–91)	67.96 (44–90)	0.69
Gender %	62% female	70% female	0.22
Average BMI (kg/m^2^)	36.1	38.3	0.10
Race			
White	88	95	0.62
Other	1	3	
ASA Class %			0.95
Class 1	5%	6%	
Class 2	45%	43%	
Class 3	50%	51%	

KOOS-JR and Knee Society scores improved significantly from their preoperative values to 6-month and 1-year follow-up visits for both 1G and 2G TKAs. Preoperatively, the average score for KOOS-JR was 30 (sd 15.6), which rose to 81 (sd 15.3) at 6 months and 84 (sd 13.9) at 1 year for 1G TKAs (p < 0.00001). The corresponding average scores for 2G TKAs were 33 (sd 11.9), 89 (sd 11.2), and 87 (sd 13.5) preoperatively, at 6 months and 1 year, respectively (p < 0.0001). Similarly, the functional score, objective knee score, patient satisfaction, and expectation scores from KSS showed similar improvements from preoperative to postoperative visits (Table 2).

**Table 2:** Preoperative, 6-month, and 1-year Knee Society component scores

**1G Objective Knee Score**				**2G Objective Knee Score**		
**Time**	**Mean**	**Sd**	**p-value**	**Mean**	**sd**	**p-value**
Pre-op	40.06	16.4		42.1	17.7	
6-month	92.6	7.2	< 0.001*	92.9	5.6	< 0.001*
1-year	94.8	5.0	< 0.001*	95.4	4.0	< 0.001*
**1G Functional Knee Score**				**2G Functional Knee Score**		
**Time**	**Mean**	**Sd**	**p-value**	**Mean**	**sd**	**p-value**
Pre-op	21.7	12.8		21.1	10.9	
6-month	69.3	18.1	< 0.001*	74.3	13.9	< 0.001*
1-year	73.3	15.8	< 0.001*	76.1	15.5	< 0.001*
**1G Patient Satisfaction Score**				**2G Patient Satisfaction Score**		
**Time**	**Mean**	**Sd**	**p-value**	**Mean**	**sd**	**p-value**
Pre-op	4.7	5.3		4.77	4.3	
6-month	33.0	7.0	< 0.001*	34.5	6.6	< 0.001*
1-year	34.5	6.4	< 0.001*	35.0	6.4	< 0.001*
**1G Patient Expectations Score**				**2G Patient Expectations Score**		
**Time**	**Mean**	**Sd**	**p-value**	**Mean**	**sd**	**p-value**
Pre-op	13.7	1.8		13.8	2.0	
6-month	10.9	2.9	< 0.001*	11.2	2.7	< 0.001*
1-year	11.9	2.8	< 0.001*	12.6	2.7	0.002*

sd = standard deviation

* statistically significant

The authors compared the outcome scores from 1G TKAs with those of 2G TKAs at each follow-up visit. Preoperatively, there were no differences between the two groups in terms of KOOS-JR, functional, knee objective, patient satisfaction and expectation scores. However, there were statistically significant differences between 1G and 2G TKAs in KOOS-JR and KSS functional scores at 6 months but not at the 1-year follow-up visit. Although the average scores of 2G at 1 year were higher, the differences did not reach significant levels (Tables 3 and 4).

**Table 3:** KOOS-JR score at 6-month and 1-year follow-up visit

		6 months			1 year	
	**Mean**	**sd**	**p-value**	**Mean**	**sd**	**p-value**
1G	80.9	15.3	< 0.001*	84.2	13.9	0.1
2G	88.7	11.2		87.4	13.5	

*statistically significant

**Table 4:** Knee Society component scores at 6-month and 1-year follow-up visits

	**Objective Knee Score**					
	**6 months**			**1 year**		
	**Mean**	**sd**	**p-value**	**Mean**	**sd**	**p-value**
1G	92.6	7.2	0.39	94.8	5	0.62
2G	92.9	5.6		95.4	4	
**Functional Knee Score**						
	**6 months**			**1 year**		
	**Mean**	**sd**	**p-value**	**Mean**	**sd**	**p-value**
1G	69.3	18.1	0.027*	73.3	15.8	0.12
2G	74.3	13.8		76.1	15.5	
**Patient Satisfaction Score**						
	**6 months**			**1 year**		
	**Mean**	**sd**	**p-value**	**Mean**	**sd**	**p-value**
1G	33.4	7	0.12	34.4	6.4	0.28
2G	34.5	6.6		35	6.4	
**Patient Expectation Score**						
	**6 months**			**1 year**		
	**Mean**	**sd**	**p-value**	**Mean**	**sd**	**p-value**
1G	10.9	2.9	0.36	11.2	2.7	0.26
2G	11.9	2.7		12.6	2.9	

sd = standard deviation * statistically significant

### Complications

Two patients (two 1G knees) had irrigation and debridement (I&D) with the exchange of polyethylene inserts due to periprosthetic joint infection at 2 and 3 months following their index TKAs. Methicillin-resistant staphylococcus aureus (MRSA) was the pathogen in one joint and streptococcus viridians in the other. Ultimately, the implant with MRSA infection was explanted 28 months after surgery. Two patients (two 2G knees) had I&D following a fall that caused open wounds. Four patients (four 1G knees) died of unrelated causes. Two patients (two 1G knees) had acute myocardial infarctions postoperatively. There were two manipulations under anesthesia for two 2G knees (two patients) 6 weeks after the surgery (p value = 0.16). One patient had a revision secondary to a distal femoral fracture after a fall, and one patient (2G knee) ruptured her quadriceps tendon 9 months after surgery, which was treated by repair of the tendon and exchange of a polyethylene insert. Twenty-one patients (eight 2G, and seventeen 1G knees) were lost to follow-up for a variety of different reasons.

## Discussion

Since the addition of total knee arthroplasty to the orthopedic surgeon’s armamentarium in the fight against debilitating degenerative knee joint disease, there have been many changes in the design of knee prostheses to improve the survival and functionality of the implants. Factors affecting patient satisfaction and performance include the design, durability, and functionality of the implants [12, 13]. Patient-reported outcome measures (PROMs) are used to assess a patient’s satisfaction and functional abilities. KOOS-JR and patient-reported components of KSS are validated, widely used PROMs that were used in this study [14, 15].

The researchers observed significant differences between 1G and 2G at 6 months in terms of KOOS-JR and functional score of KSS but not at 1 year. The scores for 2G were still higher at 1 year but not significantly. Since the cohorts were not different demographically, and the surgeon and facility were the same for both cohorts, it is reasonable to assume that the difference in scores is due to the difference in implants. The available data collected for this study suggest that design changes have resulted in an improved performance of new-generation implants in the short term. The 2G femoral component provides a more anatomic patellar tracking and better coverage with less medial-lateral overhang compared to 1G. Additionally, there is a more consistent range of contact areas across 2G implants in both the CR and PS designs. Apparently, improved replication of natural knee kinematics by 2G played a role in improving functional outcome in the short term. The authors observed that this superiority was not maintained at 1 year. This observation may imply that patients treated with the older design needed a longer time to adapt themselves to the less anatomic implant over a longer period.

One of the strengths of this study is that the patients’ demographics were not different for the two cohorts. Though there might be other factors affecting the outcome that were not investigated in the study, the main demographics of the two cohorts were no different. Also, despite the fact that majority of subjects were Caucasians, the distribution of races was similar between the two cohorts. The surgeon switched from 1G to 2G without changing the patient selection strategy or standard of care. In other words, of all of the factors affecting the outcome of a TKA, surgeon and patient factors during the study period were constant, and only the implant factor changed. One limitation of the study is the use of a single type of insert in each design cohort. Thus, the ability to compare the outcomes of different inserts was limited. Also, we were underpowered to significantly estimate the outcome differences between the CR and PS implants in each generation.

## Conclusion

In conclusion, it seems that design changes in the new generation have resulted in a product that patients rate as more functional, at 6-month follow-up, than the old generation. Though this superiority was not maintained at 1 year of follow up. Patients responded acutely to the design change as evidenced by significantly improved patient-reported outcome scores for the second generation. Future studies with a longer follow-up at more centers with added radiological outcomes would be needed to better compare the two generations of implants.

## Data Availability

The data that support the findings of this study are available from Globus Medical Inc. but restrictions apply to the availability of these data, which were used under license for the current study, and so are not publicly available.
